# Pragati: an empowerment programme for female sex workers in Bangalore, India

**DOI:** 10.3402/gha.v5i0.19279

**Published:** 2012-11-27

**Authors:** Sjoerd M. Euser, Dennis Souverein, Pushpalatha Rama Narayana Gowda, Chandra Shekhar Gowda, Diana Grootendorst, Rajendra Ramaiah, Snehal Barot, Sunil Kumar, Françoise Jenniskens, Shiv Kumar, Jeroen W. Den Boer

**Affiliations:** 1 Department of Epidemiology, Regional Public Health Laboratory Kennemerland, Haarlem, The Netherlands; 2 Swathi Mahila Sangha, Bangalore, India; 3 Swasti, A Health Resource Centre, Bangalore, India; 4 Karnataka Health Promotion Trust, Bangalore, India; 5 Françoise Jenniskens HIV and Health Advice, Haarlem, The Netherlands

**Keywords:** HIV, female sex workers, sexually transmitted infections, India, empowerment, syndromic case management

## Abstract

**Objectives:**

To describe the effects of a broad empowerment programme among female sex workers (FSWs) in Bangalore, India, which seeks to develop the capacities of these women to address the issues that threaten their lives and livelihoods.

**Design:**

This study is based on a comprehensive, on-going HIV-prevention and empowering programme, known as Pragati, which reaches out to approximately 10,000–12,000 FSWs in Bangalore each year. The programme has been designed in collaboration with the sex worker community and provides a personalised set of services, which include STI prevention and treatment services, crisis-response facilities, de-addiction services, and microfinance support all of which have been tailored to adequately fulfil each woman's needs. During the period examined by this study, the programme reached out to 20,330 individual FSWs [median (IQR) age 28 (24–35) years]. The programme's personal records of the participating FSWs were used for this descriptive study.

**Results:**

Between 2005 and 2010, the number of participating FSWs increased from 2,307 to 13,392. These women intensified their contact with the programme over time: the number of programme contacts increased from 10,351 in 2005 to 167,709 in 2010. Furthermore, data on the effects of crisis-response facilities, de-addiction and microfinance services, condom distribution schemes, and STI diagnosis and treatment showed an accumulating involvement of the participating FSWs in these programme services.

**Conclusion:**

This programme, which focuses on social and economic empowerment among FSWs, is successful in reaching and involving the target population.

India has the world's third largest population of people living with HIV with an estimated 2.4 million (0.31%) people in 2009 (1)§. The majority of HIV infections in India (87.4%) are heterosexually transmitted due to unprotected sex (1). The HIV prevalence among high-risk groups in 2009 was 9.2% for injection drugs users (IDU); 7.3% for men who have sex with men (MSM); 2.5% for people with sexually transmitted infections (STIs); and 4.9% for female sex workers (FSWs). Commercial sex is considered to be the primary driver of the HIV epidemic in India, with an estimated 1.26 million FSWs living in India (1). Within the National AIDS Control Programme (NACP), that is now in its fourth phase (2012–2017), FSWs form one of the main targets of the effort to prevent new infections by interventions such as increasing condom use and treatment of STIs (1, 2). In line with these NACP initiatives, several intervention programmes have been launched in recent years, with a focus on high-risk groups in high HIV-prevalence areas in India (2–4).

Pragati (meaning ‘progress’ in Kannada, an Indian language) is one of the leading innovative programmes, which have gone to scale in India. It is a large intervention programme among FSWs in Bangalore, the capital city of Karnataka with an estimated population of 9.59 million (Bangalore District) (5). The hypothesis of the programme is that FSWs should be socially and economically empowered to enable them to protect themselves from STIs, including HIV and AIDS, and other health threats. The main goals of the Pragati programme are to 1) reduce transmission of HIV and STIs, 2) improve the well-being of FSWs, and 3) develop the capacities of the women to protect and respond to key threats to their lives and livelihoods. The community of FSWs is organised in a sex workers collective called Swathi Mahila Sanga (SMS) and participates at all stages of the project, from conceptualisation and planning to implementation. The different activities through which the programme goals are implemented include access to drop-in centres with attached health clinics, referrals to clinics for STI and ART treatment, self-help groups for saving and credit activities, a de-addiction programme, and a crisis response mechanism for women who encounter violent behaviour.

The present article describes preliminary results of Pragati for the period between 2005 and 2010 and includes data on the performance of the different programme services.

## Methods

### The Pragati programme

The Pragati programme, which was launched in April 2005, was financially supported by the Bill and Melinda Gates Foundation, the Karnataka State AIDS Prevention Society (KSAPS), United Nations Development Programme (UNDP) (through its stigma reduction initiatives), and Vrutti (a livelihood resource centre). The programme is implemented by Swathi Mahila Sangha (SMS), a sex worker collective, in collaboration with Swasti, a health resource centre. SMS managed field operations, particularly community mobilisation and outreach activities, while Swasti was responsible for project management, financial management, organisation development of SMS, and technical support in areas such as strategy development, planning and monitoring, and evaluation. In addition, Swasti partner organization Vrutti was involved in setting up and managing the Women's Bank (Swathi Jyothi), and provided on-going technical support and capacity building. During the design phase, SMS and Swasti consulted members of the sex work community to understand their needs. These were prioritised and different services were designed to address these. Such community consultations continued throughout the programme period, with a minimum of two consultations annually. This helps to ensure that the programme continues to remain relevant to the community and continues to respond to the emerging needs of the community.

The sustainability of the Pragati programme was a key objective from the outset, recognising that a programme for FSWs needed to be driven by the women themselves. Pragati involved the community at all stages – from conceptualisation and planning to implementation, with the community leaders involved in all decision-making forums, e.g. strategic planning and monthly review meetings. A shadow leadership approach (recognised by UNDP's ‘Capacity is Development’ initiative as one of the top case studies) was adopted from the start of the programme, with the SMS staff shadowing the Swasti staff in all key positions. Swasti focused on institutional strengthening of the SMS as well as building its leadership. SMS has grown from a membership of 13 in 2005 to 6,649 members in 2010 and has a decentralised leadership model with a central board and zonal boards (all democratically elected), which has helped ensure transparency, effective governance, and transition of leadership from those who founded SMS to the emerging second line of leadership. The programme has facilitated the creation of a common platform for FSWs in Bangalore, which works on issues common to all sex workers in the city.

**Fig. 1:**
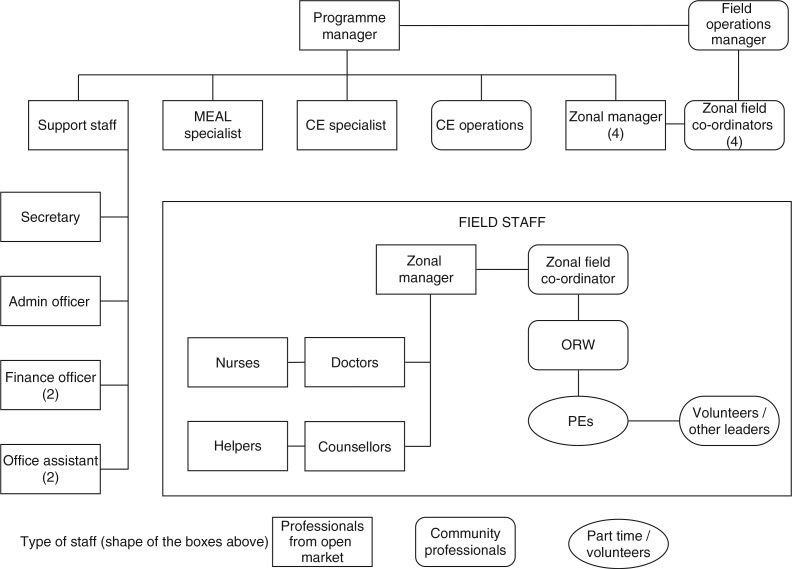
Pragati programme structure. It shows the organisation structure of the Pragati programme staff. Peer educators (PE) and outreach workers (ORW) make up the backbone of the Pragati project at field level. Each PE is responsible for 60–120 FSWs to whom she regularly provides outreach services and encourages visiting the Pragati drop-in centres. These PEs are supported by an ORW, who is responsible for day-to-day monitoring of the PEs, providing them handholding support when required, establishing condom outlets, and the management of secondary stakeholders in her assigned area. Each ORW is responsible for 6–7 PEs. The ORWs report to zonal field coordinators who are from the sex worker collective. A zonal manager supports the field coordinators. There is an overall field operations manager from the sex worker collective (Swathi Mahila Sangha) and a programme manager from the technical support agency (Swasti Health Resource Center) who jointly oversee the project. MEAL = monitoring, evaluation and learning.

The main goal of the programme is to reduce HIV and STI transmission among FSWs and improve their well-being by developing their capacities. The programme is categorised into three broad areas of intervention: 1) *protect and respond*, which involves implementing an outreach strategy where peer educators and outreach workers start a dialogue with the women about the services and benefits offered through the programme. This strategy also involves sensitising primary and secondary stakeholders of the sex worker industry (such as brothel owners, pimps, and the police) to problems and needs of FSWs. 2) *Improve their quality of life* through identifying and addressing long-term development needs such as support for alcohol de-addiction, provision of saving and credit facilities, and creating options for alternative and diversified livelihoods. 3) *Build the capacities* of the women to address the issues that threaten their lives and livelihoods through strengthening group action and developing a strong collective of sex workers. An extensive explanation of the programme structure is shown in Fig. 1.

### Empowerment strategy

The Pragati programme followed an empowerment approach (largely drawn from the work of Dr. Srilatha Batliwala (6), which places the women at the centre and seeks to address the internal and external factors that affect their vulnerability to HIV and STIs. The internal factors were the women's own beliefs, myths, and misconceptions; their low levels of confidence and capacity; lack of ability to bargain collectively; and their unquestioning acceptance of established power and social structures. The external factors were the violence and harassment afflicted upon the women, discrimination caused by social structures, and the legal framework within which the sex work industry operates. Taking into account the above factors, the programme set out to empower the women to 1) improve their ability to address their own well-being; 2) make informed choices; 3) access and control resources and; and 4) realise their rights. To achieve these overall outcomes, the programme focused on ways to improve the women's self-consciousness and confidence; build their capacities; strengthen individual and collective action; and reduce violence and harassment experienced by the women. The strategies used included capacity development, representation and democratisation, fostering leadership, creative advocacy, and alliance-building with stakeholders.

### Programme setting

In 2004, the Karnataka State AIDS Control Society in collaboration with key NGOs and community-based organisations (CBO) led a mapping exercise in Bangalore to estimate the number of FSWs, thereby following the city's administrative division into seven zones. Two of the seven zones (zone 2 and 5) were already covered by targeted HIV interventions led by other NGOs/CBOs. Following the mapping, the Pragati programme was initiated in zone 1 and 3. These two zones were initially selected as SMS had been previously operating here. In November 2005, this was expanded to include zone 4 and 6. Zone 7 was not seen as a priority in 2005 as it had a small number of sex workers. As the number increased over the years, a separate state-funded intervention was initiated there in November 2008.

### Participants

Between April 2005 and October 2010, all FSWs who solicited sex in the four selected zones out of the designated seven in the city of Bangalore were eligible for participation in the Pragati programme. These women were approached by peer educators and outreach workers (all former or current FSWs) of Pragati at the FSWs soliciting areas in the city. They motivated the FSWs to visit the drop-in centres by explaining what services are provided there. Within the four programme zones of Bangalore, several drop-in centres were accessible to offer the FSWs a safe place to rest and relax, take a shower, have a meal, meet peers, and to get in contact with the Pragati staff. During these contacts, FSWs were informed about the different services and areas of support available for them. Upon registration, data on socio-economic status and sex work details of the participating FSWs were obtained. Subsequently, the women could participate in the various activities of Pragati.

### Pragati project activities

The various activities of Pragati range from social and economic empowerment tools like crisis-response teams that try to diminish the amount of violence and harassment that FSWs face, and financial support by microfinance systems, to health-related components such as de-addiction programmes, condom use promotion, and STI prevention and treatment strategies. Services at the drop-in centres included provision of information and counselling on HIV and AIDS; demonstration of correct and consistent condom use; distribution of free and social marketed male and female condoms; HIV and syphilis tests; de-addiction programmes; facilities for taking a bath, sleep, and entertainment; provision of relevant legal information; and saving as well as credit facilities through a microfinance institution, exclusively for FSWs. Data on services provided at the drop-in centres were recorded. When experiencing health problems, women could visit one of the participating clinics (programme-linked clinics or private referral clinics). Inside each drop-in centre, a clinic was organised where medical staff were present to provide health-related counselling, medical examinations, and treatment when needed.

### Violence redressal

FSWs are easy targets for violence and harassment. One of the primary concerns of the women identified during the planning phase of the project was violent behaviour towards them by perpetrators ranging from clients, fellow sex workers, police, criminals, regular partners (not clients or husbands), to lovers and husbands. A violence redressal mechanism was established within Pragati to address issues related to violence, harassment, and trafficking. A rapid response team of women in sex work is now available (24/7) to extend timely medical, legal, and psycho-social support to the victims. This mechanism (called Swathi Nyaya Sanjeevani) educates the FSWs on their legal rights and strives to empower the women to defend themselves. It also sensitises the perpetrators of violence (such as police, criminals, pimps, and brokers in the sex working industry), on issues related to FSWs, and promotes awareness of women's rights among them. Swathi Nyaya Sanjeevani takes proactive steps to prevent violence by identifying volatile regions and establishing violence watch committees for vigilant monitoring of warning signs.

### Alcohol de-addiction

Consumption of alcohol has led to many FSWs becoming alcohol dependent. When intoxicated, the women are frequently not in a strong position to insist on the use of condoms, which accentuates their risk for STI and HIV infection. Under the influence of alcohol, many women are also harassed by their clients and peers. Swathi Chetana facilitates alcohol de-addiction among women. This programme ensures that the women undergo de-addiction counselling, promotes the reduction in alcohol consumption, and motivates the women to give up alcohol. Additionally, Swathi Chetana has established linkages with the local government hospital. The hospital facilitates initial consultations with a psychiatrist or a psychologist.

### Microfinance

Financial constraints are one of the precipitating factors for women to enter sex work. FSWs struggle not only with earning a decent income, but also with ensuring that the money is kept safely away from violent perpetrators who steal it from them. Many women do not have documented proof of identity; some even live in the streets. Such barriers prevent the women from accessing mainstream financial services. At times, the women take loans from non-mainstream financial services (such as money lenders) who charge exorbitant interest rates. Financial insecurity can also make them fall prey to the lures of clients who offer a better payment for sex without condoms, which increases their vulnerability to STI and HIV infection. Pragati facilitated the formation of Swathi Jyothi Mahila Vividhodesha Souhardha Sarkari Niyamita, a microfinance institution, which offers a savings bank, recurring deposit, and term loan services to the members. This institution is managed as a cooperative bank and owned by the FSWs. Women who are registered with Pragati and have membership in SMS can open a savings account and walk in to any of the drop-in centres to deposit money into their own accounts.

### Promotion of condom use

Through communication for behavioural change as well as the distribution of condoms, Pragati promotes correct and consistent condom use among the FSWs. Every woman registered in the project is assessed to understand whether she has heard about, seen, or ever used condoms. The outreach team and counsellors provide demonstrations to familiarise the women with condom use and to emphasise the importance of consistent and correct use of condoms. Information on the various brands of condoms is given, and after assessing the client volume, women are provided with the required number of condoms. The programme has also established condom stock points at hotspots and maintains a distribution system to replenish it and thereby ensures that women have easy access to condoms when they need them.

### Sexually transmitted infections

The FSWs who experience health problems, can visit one of the clinics in the Pragati programme for a medical examination and treatment. In these clinics, STIs are diagnosed using syndromic case management, in line with the methods suggested in the WHO guidelines (7), and implemented in India according to National AIDS Control Organisation (NACO) guidelines (8, 9). Reported syndromes of STIs included genital ulcers, inguinal swellings, vaginal discharge, cervicitis, and lower abdominal pain. All symptoms of the same STI, which were reported within 7 days of a previous visit (14 days for vaginitis, 21 days for inguinal swelling), were considered as the same episode of that STI.

### STI treatment

All women presenting with STI symptoms received immediate treatment in conformity with the syndromic case management guidelines issued by NACO (7–9). Irrespective of the presence of symptoms, each woman who visited any of the participating clinics for the first time or whose last visit was more than 3 months (April 2005 to March 2010) or 6 months (from April 2010 onwards) ago, received presumptive treatment for asymptomatic infections. The treatment regimens are presented in Table 1.

**Table 1:** STI treatment packs

Treatment pack	STI symptoms	Medication
Grey-1	Cervicitis	Azithromycin 1g tablet and cefixime 400 mg
White	Genital ulcers non-herpes	Benzathine penicillin 240.000 units intramuscular injection with azithromycin 1g tablet stat or twice daily ciprofloxacin 500 mg for three days*
Red	Genital ulcers herpes	Acyclovir 400 mg orally 3 times daily for 7–10 days
Black	Inguinal swelling	Doxycycline 100 mg twice daily for 21 days
Green	Vaginitis	Metronidazole 2g tablet or tinidazole 2g tablet and 150mg fluconazole
Yellow	Pelvic inflammatory disease (PID)	Ceftriaxone 250 mg intramuscular injection together with doxycycline100 mg twice daily for 14 days and metronidazole 400 mg twice daily for 14 days
Grey-2	Asymptomatic infections	Azithromycin 1g tablet and cefixime 400 mg and metronidazole 2g tablet or tinidazole 2g tablet

* For persons allergic to penicillin, 14 days doxycycline 100 mg and azithromicin 1G tablet is administered. For pregnant women, 15 days erythromycin stearate 500 mg and azithromycin 1G tablet is administered.

### Statistical analysis

Data were analysed using descriptive statistics and are presented as mean with standard deviation, median with inter quartile range (IQR), or frequency, as appropriate. Follow-up time was calculated from the date of the first contact to the date of the last contact and expressed in years. Person-time was calculated as the sum of follow-up time and expressed in person-years. Contact rates were calculated as the sum of contacts with the programme divided by the sum of person-years. Incidence rates of STIs were calculated as the sum of STI episodes divided by the sum of person-years. Both contact rates and incidence rates were stratified by year in order to examine trends over time. All analyses were performed with PASW Statistics release 18.0, SPSS Inc., Chicago, Illinois.

## Results

### Participants

Between 1 January 2005 and 31 October 2010, a total of 20,330 FSWs were enrolled in Pragati (Table 2). Their median age (IQR) was 28 (24–35) years, and the educational level was relatively low with almost half of the participants (49.4%) being illiterate. The participating FSWs were evenly distributed over the four zones of Bangalore, which are covered by the programme. Most FSWs solicited for sex work at home (39.9%); on the street, in parks, or other public places (30.5%); or in a rented room (15.9%). Nearly half of the FSWs (48.3%) were married, 10.0% were widowed, and 23.7% were divorced, separated from, or deserted by their husband. In 2005, when Pragati had just started, data from 2,307 FSWs were recorded during their first contact with the programme. This number increased to 10,751 FSWs in 2006 and to 13,932 FSWs who had been in contact with the programme in 2010.

**Table 2:** Characteristics of participating female sex workers over time (*n =* 20,330)

		Year					
		
Characteristics	Total	2005	2006	2007	2008	2009	2010
Number	20,330	2,307	10,751	14,637	16,606	14,555	13,932
Median age, yrs (IQR)	28 (24–35)	29 (24–35)	29 (24–35)	29 (24–35)	29 (34–35)	29 (24–35)	28 (24–35)
Age group†							
10–23 yrs	4,828 (23.8%)	547 (23.9%)	2,474 (23.1%)	3,353 (23.0%)	3,762 (22.8%)	3,294 (22.7%)	3,234 (23.3%)
24–28 yrs	5,407 (26.7%)	555 (24.2%)	2,786 (26.0%)	3,827 (26.3%)	4,449 (26.9%)	3,955 (27.3%)	3,821 (27.5%)
29–34 yrs	4,176 (20.6%)	470 (20.5%)	2,192 (20.5%)	2,982 (20.5%)	3,447 (20.8%)	3,037 (20.9%)	2,892 (20.8%)
35+ yrs	5,834 (28.8%)	720 (31.4%)	3,245 (30.3%)	4,411 (30.3%)	4,877 (29.5%)	4,213 (29.1%)	3,933 (28.3%)
Education‡							
Illiterate	9,035 (49.4%)	1,013 (47.6%)	4,490 (49.1%)	6,559 (51.1%)	7,579 (50.9%)	6,624 (50.3%)	6,246 (49.3%)
Literate but not been to school	77 (0.4%)	6 (0.3%)	29 (0.3%)	43 (0.3%)	64 (0.4%)	58 (0.4%)	60 (0.5%)
1–4 standard completed	2,278 (12.5%)	193 (9.1%)	954 (10.4%)	1,369 (10.7%)	1,802 (12.1%)	1,683 (12.8%)	1,698 (13.4%)
5–7 standard completed	3,259 (17.8%)	401 (18.9%)	1,718 (18.8%)	2,247 (17.5%)	2,569 (17.2%)	2,327 (17.7%)	2,264 (17.9%)
8–10 standard completed	2,989 (16.4%)	429 (20.2%)	1,623 (17.7%)	2,172 (16.9%)	2,396 (16.1%)	2,045 (15.5%)	1,973 (15.6%)
11 standard and above	636 (3.5%)	84 (4.0%)	336 (3.7%)	450 (3.5%)	485 (3.3%)	426 (3.2%)	416 (3.3%)
Soliciting place††							
Home	7,064 (39.9%)	274 (16.3%)	2,797 (32.7%)	4,489 (35.8%)	5,929 (40.8%)	5,311 (40.9%)	5,161 (41.1%)
Rented room	2,814 (15.9%)	232 (13.8%)	1,775 (20.7%)	2,315 (18.5%)	2,302 (15.8%)	2,010 (15.5%)	1,836 (14.6%)
Lodge	851 (4.8%)	150 (8.9%)	582 (6.8%)	687 (5.5%)	468 (2.7%)	545 (4.2%)	524 (4.2%)
Brothel	1,007 (5.7%)	66 (3.9%)	382 (4.5%)	603 (4.8%)	726 (5.0%)	664 (5.1%)	727 (5.8%)
Street/Park/Public	5,410 (30.5%)	851 (50.7%)	2,689 (31.4%)	3,926 (31.3%)	4,398 (30.2%)	4,039 (31.1%)	3,908 (31.1%)
Other*	569 (3.2%)	105 (6.3%)	341 (4.0%)	519 (4.1%)	504 (3.5%)	432 (3.3%)	410 (3.3%)
City zone							
Zone 1	4,666 (23.0%)	926 (40.1%)	2,626 (24.4%)	3,392 (23.2%)	3,639 (21.9%)	3,228 (22.2%)	3,209 (23.0%)
Zone 3	5,189 (25.5%)	1,207 (52.3%)	3,052 (28.4%)	3,833 (26.2%)	3,957 (23.8%)	3,120 (21.4%)	2,941 (21.1%)
Zone 4	5,243 (25.8%)	2 (0.1%)	2,276 (21.2%)	3,475 (23.7%)	4,582 (27.6%)	4,129 (28.4%)	3,880 (27.8%)
Zone 6	5,232 (25.7%)	172 (7.5%)	2,797 (26.0%)	3,937 (26.9%)	4,428 (26.7%)	4,078 (28.0%)	3,902 (28.0%)

Data are numbers (%) unless indicated otherwise.

† Data were available for 20,245 women;

‡ data were available for 18,274 women;

†† data were available for 17,715 women.

* Included bar/night club, vehicle, theatre, and other soliciting places.

### Contacts

**Fig. 2:**
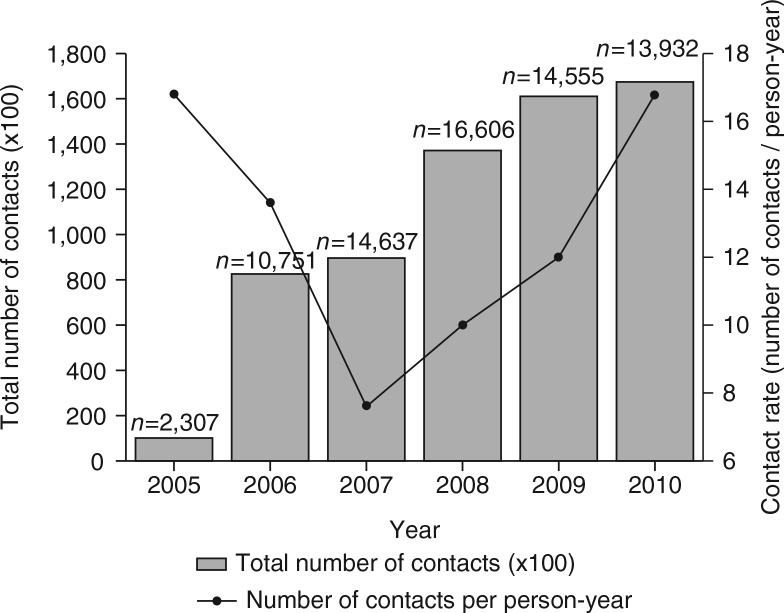
Contacts with the programme over time. Bars represent the total number of contacts per year. Line represents the contact rate over time (contacts/person-year).

In total, 649,438 contacts with the programme were recorded for the 20,330 FSWs between 2005 and 2010 (Table 3, Fig. 2). These contacts included peer educator contacts, contacts with programme clinics, and contacts with referral clinics. At the start of the programme in 2005, four drop-in centres were opened as well as four programme clinics. After 4 years, in 2009, there were eight drop-in centres, eight programme clinics, and 90 referral clinics available for the participating FSWs. In 2010, four drop-in centres, four programme clinics, and 80 referral clinics were available. The total number of contacts with the programme increased from 10,351 contacts in 2005 to 167,709 contacts in 2010, which was primarily driven by the peer educator contacts. The contact rate for programme clinics declined between 2005 and 2010, while the contact rate for referral clinics increased during this period (Table 3).

**Table 3:** Programme contacts over time

			All contacts		Peer–education contacts		Project clinic contacts		Referral clinic contacts		Other contacts*	
							
Year	Number of FSWs	Person–years	Number of contacts	Rate	Number of contacts	Rate	Number of contacts	Rate	Number of contacts	Rate	Number of contacts	Rate
2005	2,307	616.9	10,351	16.8	7,555	12.2	1,680	2.7	0	0	1,116	1.8
2006	10,751	6077.2	82,819	13.6	61,341	10.1	11,183	1.8	7	0.001	10,288	1.7
2007	14,637	11843.7	89,862	7.6	54,057	4.6	24,190	2.0	264	0.002	11,351	1.0
2008	16,606	13675.5	137,401	10.0	102,889	7.5	20,934	1.5	5,836	0.4	7,742	0.6
2009	14,555	13492.0	161,296	12.0	125,565	9.3	8,001	0.6	24,784	1.8	2,946	0.2
2010	13,932	9990.3	167,709	16.8	132,877	13.3	5,498	0.6	24,323	2.4	5,011	0.5
Total	72,788	55695.6	649438	11.7	484284	8.7	71,486	1.3	55,214	1.0	38,454	0.7

Contact rates reflect the number of contacts per person-year.

* Contains registration and follow-up (for syphilis and HIV testing) contacts.

### Violence redressal

**Fig. 3:**
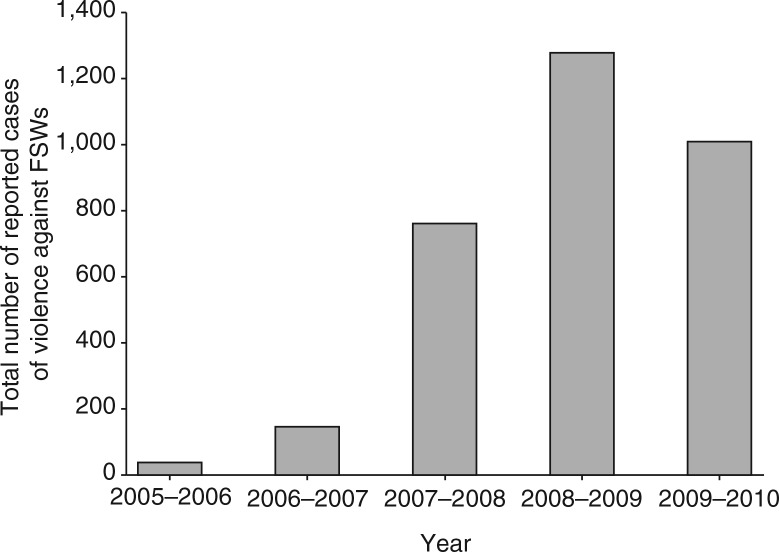
Reported cases of violence against FSWs per year (2005–2010). Bars represent the total number of reported cases of violence against FSWs per year. Reporting period was 1 April–30 March of the next year.

During the period 2005–2010, Swathi Nyaya Sanjeevani dealt with a total of 3,242 reported cases related to violence against FSWs in Bangalore. The annual number of reported cases increased from 40 cases in 2005–2006; 148 cases in 2006–2007; 763 cases in 2007–2008; 1280 cases in 2008–2009; to 1011 cases in 2009–2010 (Fig. 3). The reporting period was 1 April–30 March of the next year. In most cases, a response was initiated within 24 hours. The majority of the reports were related to violence by the police (37%). The other major perpetrators were partners (14%), other FSWs (12%), and husbands (9%).

### Alcohol de-addiction

During a period of 5 years (April 2005–March 2010), 1,377 FSWs were identified and referred for alcohol de-addiction. Of those women, 433 underwent treatment. The participation in the alcohol de-addiction programme increased from 0 women in 2005–2006; 21 women in 2006–2007; 110 women in 2007–2008; 71 women in 2008–2009; to 231 women in 2009–2010 (reporting period was 1 April–30 March of the next year).

### Microfinance

There were in total 3,053 FSWs who joined as shareholders of the cooperative bank during 2007–2010. More than half of them (52%; 1578 individuals) opened savings accounts. The number of women who opened a savings account increased from 488 women in 2007–2008; 501 women in 2008–2009; to 589 women in 2009–2010 (reporting period was 1 April–30 March the next year). Over 1,700 women opened recurrent deposit accounts. Total savings through various schemes of the bank during 2007–2010 accumulated to 2.2 million rupees (approximately 38,500 USD). The total amount of loans that were distributed to 406 women equalled 4.5 million rupees (approximately 79,000 USD).

### Condom distribution

**Fig. 4:**
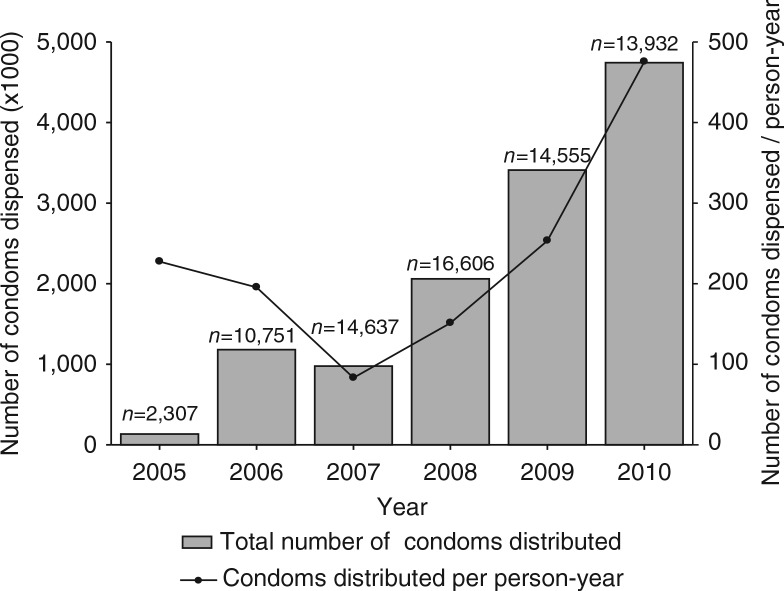
Condoms distributed per year (2005–2010). Bars represent the total number of distributed condoms per year. Line represents the number of condoms distributed per person-year.

The number of distributed condoms increased sharply between 2005 and 2010, with 140,248 condoms distributed in 2005 to 4,748,943 condoms distributed in 2010 (Fig. 4). Additionally, the number of condoms distributed per person-year also increased over time, from 227 condoms/person-year in 2005 to 475 condoms/person-year in 2010 (Fig. 4). In 2010, across four zones, 285 condom outlets catered to the requirement of women in sex work.

### Sexually transmitted infections

**Fig. 5:**
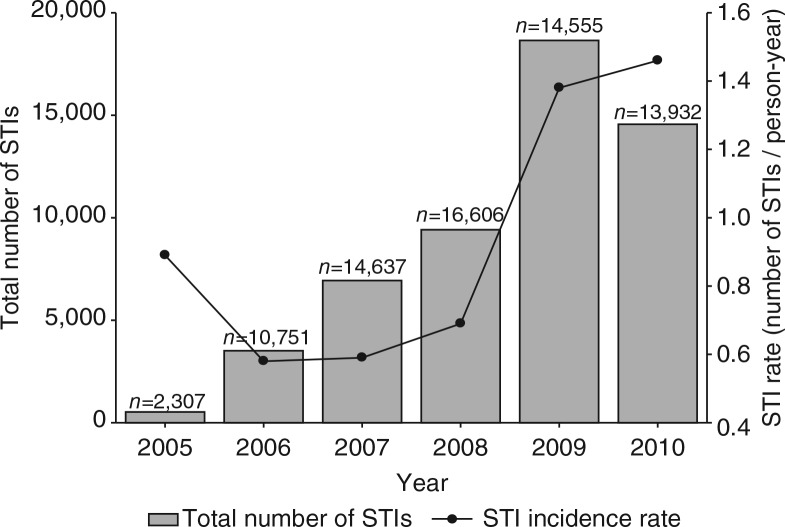
Diagnosed STIs and STI incidence rate over time. Bars represent the total number of diagnosed STIs per year. Line represents the STI incidence rate over time (STI/person-year).

Overall, there were 53,762 episodes of STIs recorded between 2005 and 2010, for 14,162 individual FSWs. This resulted in a mean STI incidence rate of 0.97 STIs/person-year (Fig. 5). The most frequently diagnosed STIs were cervicitis (50.6% of the total STIs) and vaginitis (41.4%) (Table 4).

**Table 4:** Number of diagnosed STIs over time

	All STIs	Vaginitis	Cervicitis	PID	Genital ulcer (NH)	Genital ulcer
2005	549	208 (37.9)	307 (55.9)	31 (5.6)	0 (0)	3 (0.5)
2006	3,542	1,456 (41.1)	1,718 (48.5)	306 (8.6)	33 (0.9)	29 (0.8)
2007	6,963	2,467 (35.4)	3,790 (54.4)	517 (7.4)	109 (1.6)	80 (1.1)
2008	9,445	3,505 (37.1)	5,094 (53.9)	592 (6.3)	145 (1.5)	109 (1.2)
2009	18,675	7,750 (41.4)	9,676 (51.8)	1,015 (5.4)	91 (0.4)	143 (0.8)
2010	14,588	6,886 (47.2)	6,633 (45.5)	953 (6.5)	51 (0.3)	65 (0.4)
Total	53,762	22,272 (41.4)	27,218 (50.6)	3,414 (6.4)	429 (0.8)	429 (0.8)

### STI treatment

**Fig. 6:**
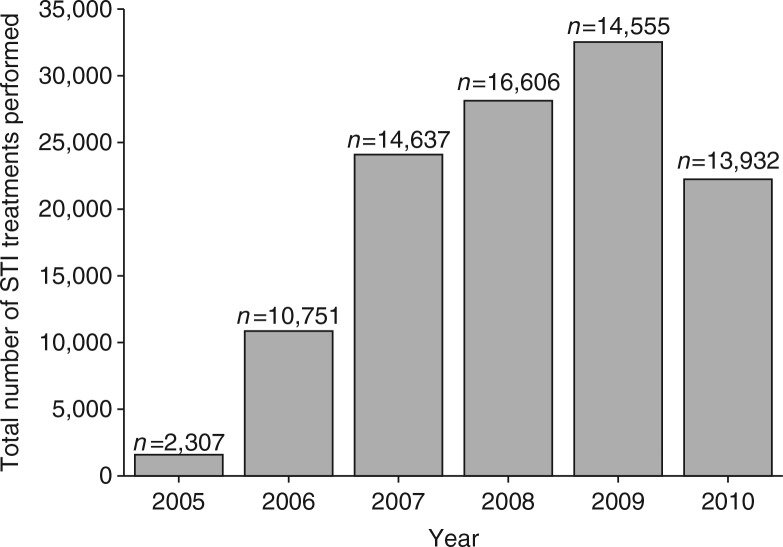
Distributed STI treatment packages over time. Bars represent the total number of STI treatment packages that were distributed per year.

The distribution of treatment ‘packs’ for the diagnosed STIs increased over time (Fig. 6) from 1,638 distributed packs in 2005 to 22,283 packs in 2010.

### Cost efficiency

The Pragati programme through effective leveraging of resources, partnerships, and achieving scale has been cost-efficient. The programme cost per participating sex worker per year of Pragati during 2005–2010 was approximately INR 2,017 (US$38), which is comparable to INR 2,000 (U$38) per sex worker as prescribed by the guidelines of NACO (10). However, the targeted interventions among FSWs under the national programme were HIV prevention centric with limited efforts towards violence reduction and collectivisation. Pragati, at similar costs, offered a more comprehensive set of HIV vulnerability reduction activities such as services (i.e. microfinance, violence redressal and reduction); anti-trafficking activities; social protection; alcohol de-addiction; individual, group, and institutional capacity building. More importantly, the cost per participating sex worker in the Pragati declined between 2005 and 2010 as the programme's scale increased: the cost per participating sex worker (reporting period 1 April–30 March of the next year) was INR 2,992 (US$56) in 2005–2006; INR 2,142 (US$40) in 2006–2007; INR 2,429 (US$46) in 2007–2008; INR 1,672 (US$32) in 2008–2009; and INR 1,628 (US$31) in 2009–2010.

## Discussion

Overall, this study has shown that the Pragati programme has had a beneficial effect on the lives and livelihoods of the 20,330 participating FSWs in Bangalore, India. Between 2005 and 2010, the number of women who received help from a crisis-response team increased, more women participated in alcohol de-addiction programmes, and the number of saving accounts and distributed microfinance loans was expanded. Furthermore, condom use increased over time, and more FSWs were treated for STIs. In contrast to these findings, the number of STIs and the STI incidence rate increased over time.

There have been several studies that indicate the disproportionate burden of HIV in FSWs compared to other women (11), and these studies have investigated the effectiveness of different intervention programmes (12, 13). However, to our knowledge, this study is the first showing longitudinal follow-up in a large group of FSWs in India and reporting data of the empowering strategy on such a wide range of social, economic, and health-related variables. Previous studies that analysed the effect of behavioural interventions on STIs primarily focussed on interventions to change sexual behaviour, promote condom use, and educate about effective management of STIs (14, 15). Although these interventions appeared to be effective in reducing STI prevalence and incidence, it was also suggested to further explore other, more potent behavioural change strategies that might show even more favourable results (13–16).

### Study limitations

The present study has limitations. First, the Pragati programme is an observational registration of 20,330 FSWs in four zones of Bangalore. Components of the programme were developed and ranked in priority in discussion with the participants. As such, different components were implemented while the programme was on-going, based on the needs of the women. This could complicate the analyses of the effectiveness of these components in the 1st years of the enrolment of the programme. Second, STIs were diagnosed according to syndromic case management. Therefore, trends in specific causes of STIs are unavailable. However, syndromic case management is more accurate than diagnosis based on clinical judgment alone, even in experienced hands, and more cost-effective for some syndromes than the use of laboratory tests (7). A major advantage of syndromic case management in populations such as FSWs is that with the use of flowcharts to guide toward diagnosis, treatment can be provided immediately at first contact, thereby reducing secondary cases and infectiousness (17). Although it has been shown that there is a wide variation between self-reporting of morbidity and syndromic- and aetiology-based diagnosis of STIs (18), syndromic case management remains the best available option in low-income settings when diagnostic testing facilities are unavailable for high-risk populations like FSWs. Finally, it would have been interesting to analyse the proportion of FSWs who had been able to create options for an alternative livelihood and leave the commercial sex-work business, as a result of participation in the Pragati programme. Unfortunately, no follow-up data were available for FSWs who had left the programme.

### Interpretation of the results

How can our results be explained? Most findings of the present analysis are in line with the increased empowerment of the participating FSWs. First, the number of contacts with the programme increased over time, suggesting social empowerment of the participating women. In the first 3 years (2005–2007), a decrease in the contact rate was seen, which was followed by an increase in the last years of the study period (2007–2010). A possible explanation is that the FSWs who were enrolled in the programme in the first years were a motivated selection of all 20,330 participating FSWs and had an above average number of programme contacts. The relatively small number of FSWs who were present in the programme in 2005 (*n*=2,307) and 2006 (*n =* 10,751) could have resulted in an inflated contact rate in these years. From 2007 onwards, the annual number of participating FSWs was on average about 15,000 and the contact rate showed a steady increase from that moment onwards. Second, the FSWs in Pragati increasingly used the available crisis-response mechanism and the violence watch committees between 2005 and 2010, which shows the need for these facilities. Third, the alcohol de-addiction programme showed a sharp increase in participating women within the study period. In the absence of success rates of the de-addiction programme, we still may assume that it has most likely improved the social as well as economic functionality of the women who successfully completed the treatment. Not only has it helped the women to overcome their addiction, a supplementary effect is that this has probably reduced their odds of exposure to HIV infection by ensuring correct and consistent condom use during sex. Unfortunately, no follow-up data were available on the sustainable effects of the de-addiction programme, which complicates the interpretation of these findings. Fourth, the microfinance mechanism has helped over 1,500 women in sex work to open a savings or deposit account during the study period, which helped to provide greater financial security. Fifth, the condom distribution showed a steady increase during the study period, which could be an example of a favourable change in sexual behaviour of the FSWs. Finally, the STI incidence rate that was seen between 2005 and 2010 seems to imply a reverse effect of the programme efforts. Our hypothesis is that that the increase in episodes of STI is not a ‘true’ increase in incidence of STI episodes but rather a consequence of detection bias: the enhanced empowerment with more regular check-ups in the clinic allowed more timely diagnosis of STIs at a less severe stage. This presumed decrease in the under-diagnosis and under-treatment of STIs could be overshadowed by the findings on the increasing STI incidence rate, but could well be another achievement of the programme. It will be interesting to see if the trend reverses in future analyses of the programme, as other studies imply that a longer follow-up period is needed (4, 19) (20). Although the presented data indicate that the Pragati programme has succeeded in establishing a multifaceted empowering programme for FSWs, additional analyses focussed on the effectiveness of the discrete programme activities on outcome measurements are necessary to further clarify their role in improving the well-being of the participating FSWs. Furthermore, it might be interesting to evaluate if certain subgroups of FSWs are differentially susceptible for the effects of the Pragati activities.

## Conclusion

In conclusion, our results show that a programme focused on social and economic empowerment among FSWs, combined with medical treatment, is successful in reaching and involving the target population. Women frequently used the facilities offered in drop-in centres and clinics involved in the programme. Future studies and analyses should focus on the evaluation of different aspects of empowerment programmes and the effects.

## Footnotes

§
Meaning ‘progress’ (in Kannada – an Indian language)
